# Efficacy of Polyvinylpyrrolidone–Zinc Gluconate and Taurine Gel in the Prophylaxis of Oral Mucositis in Adults Undergoing High-Dose Chemotherapy and Allogeneic Stem Cell Transplantation

**DOI:** 10.3390/diseases13120408

**Published:** 2025-12-18

**Authors:** Gaetana Porto, Annalisa Pitino, Mercedes Gori, Martina Pitea, Maria Eugenia Alvaro, Giovanni Luigi Tripepi, Giorgia Policastro, Fortunata Martino, Rosalba Daniela Minniti, Jessyca Germano’, Barbara Loteta, Giovanna Utano, Erica Bilardi, Francesca Cogliandro, Caterina Alati, Violetta Marafioti, Graziella D’Arrigo, Massimo Martino

**Affiliations:** 1 Hematology and Stem Cell Transplantation and Cellular Therapies Unit (CTMO), Department of Hemato-Oncology and Radiotherapy, Grande Ospedale Metropolitano “Bianchi-Melacrino-Morelli”, 89133 Reggio Calabria, Italy; 2 Institute of Clinical Physiology (IFC-CNR), Section of Rome, 00185 Rome, Italy; 3 Division of Hematology, Department of Human Pathology in Adulthood and Childhood “Gaetano Barresi”, University of Messina, via Consolare Valeria, 98125 Messina, Italy; 4 Institute of Clinical Physiology (IFC-CNR), Section of Reggio Calabria, 89124 Reggio Calabria, Italy

**Keywords:** polyvinylpyrrolidone-zinc gluconate, taurine gel, oral mucositis, high-dose chemotherapy, allogeneic stem cell transplantation, RIC, MAC

## Abstract

Background: Oral mucositis (OM) is a significant complication after allogeneic stem cell transplantation. Objectives: This prospective, observational cohort study assessed the effectiveness of a polyvinylpyrrolidone-zinc gluconate and taurine (PVP-ZG-TAU) oral gel in managing OM. The primary objective was to determine whether the gel reduced the incidence and grade of OM and accelerated its resolution. Methods: The study enrolled 82 patients; 39 received the PVP-ZG-TAU gel, and 43 represented a historical control group. To prevent oral mucositis, both groups maintained good oral hygiene. In the experimental group, patients received three sprays of PVP-ZG-TAU gel, three times a day, from the start of conditioning chemotherapy until day +15 after allo-SCT. Results: In the PVP-ZG-TAU group, 79.1% patients experienced grade 1–2 OM and 20.9% experienced grade 3–4 OM. In the control group, 74.4% had grade 1–2 OM, and 25.6% had grade 3–4 OM (*p* = ns). Resolution occurred significantly faster in the PVP-ZG-TAU group, with an 84% resolution rate per 100 person-weeks, compared with 62% in the control group. Cox regression analysis revealed that treatment was associated with a 68% greater likelihood of earlier resolution (adjusted hazard ratio [HR], 1.68; 95% confidence interval [CI], 1.03–2.74; *p* = 0.036). Conclusions: These findings suggest that PVP-ZG-TAU can reduce OM duration and serve as a supportive intervention for allo-SCT patients.

## 1. Introduction

Oral mucositis (OM) is a common complication of allogeneic stem cell transplantation (allo-SCT). It affects up to 75% of patients who receive high-dose chemotherapy (HDC) conditioning regimens [1,2]. OM is associated with pain, eating and swallowing difficulties, and the need for enteral or parenteral nutrition. It increases opioid consumption [3]. In immunosuppressed patients, OM is also tied to bacteremia, more extended hospital stays, and higher mortality within 100 days post-transplant [4]. OM develops from cytostatic agents that act on the oral epithelium, as well as from impaired immune function and reduced salivation. Regeneration of damaged epithelial cells is disrupted during anticancer therapy. Oral erosions and ulcers may develop, serving as entry points for viral, fungal, and bacterial antigens [5,6,7]. Progress in reducing OM’s burden has been minimal. Patients often see OM as the worst symptom affecting quality of life [8]. There is even less clinical evidence for prophylaxis in allo-SCT patients. Advanced-stage mucositis frequently needs intensive care. OM may disrupt patients’ food and fluid intake, extend hospitalization, and impact treatment response [9]. More patients are undergoing allo-SCT, as reduced-intensity conditioning now allows older adults and those with other diseases to receive treatment [10,11].

Current management includes preventive oral cryotherapy during cytotoxic agent administration and supportive care, such as pain management with narcotic analgesics, nutritional support, and oral hygiene to prevent infections [12,13,14]. An approach to preventing mucositis in allo-SCT is the use of polyvinylpyrrolidone (PVP) combined with zinc gluconate (ZG) [15,16]. PVP is a high-molecular-weight polymer that coats tissues and forms a permeable film. ZG is a mineral salt, while taurine (TAU) is an amino sulfonic acid. Together, ZG and TAU form a bioactive zinc-taurine complex under physiological conditions. The PVP-ZG-TAU complex demonstrates strong bacteriostatic activity without the cytotoxicity of some preservative systems [17]. The hypothesis is that combining physical barrier protection (PVP) with biological healing acceleration (Zinc) addresses mucositis through complementary mechanisms across all pathophysiologic phases, potentially preventing/reducing severity beyond current reactive approaches. PVP-ZG represents rational multi-mechanistic prevention (barrier + healing), addresses all mucositis phases, uses safe/available agents, and targets a complication with a massive clinical/economic impact for which current prevention fails most patients. Most mucositis prevention studies have been conducted in pediatric populations, leaving adult allo-SCT patients, who comprise the majority of transplants, without age-specific evidence to guide prophylaxis. The study aimed to evaluate the efficacy of PVP-ZG-TAU in reducing mucositis duration in adult patients receiving HDC and allo-SCT.

## 2. Materials and Methods

This longitudinal, real-world, double-arm study compared PVP-ZG-TAU oral gel (OG) to a control group. Eligible patients were aged 18 to 70 years. They had acute myeloid leukemia (AML), acute lymphoblastic leukemia (ALL), or myelodysplastic syndrome (MDS) in first- or later-onset hematologic complete remission (CR) according to international classifications [18,19]. Allo-SCT eligibility included a Karnofsky Performance Status (KPS) of at least 70% for standard conditioning and 60% for reduced-intensity conditioning (RIC). Other criteria were ECOG Performance Status 0–2 (0–3 for RIC); LVEF ≥ 45%; no symptomatic coronary artery disease; ECG without significant arrhythmias; FEV1 and FVC ≥ 50% predicted value; DLCO ≥ 40–50% of predicted value (adjusted for hemoglobin); PaO2 ≥ 70 mmHg on room air; total bilirubin ≤ 2.5× upper limit of normal; no active hepatitis or cirrhosis; and creatinine clearance of 50–60 mL/min or serum creatinine ≤ 2.0 mg/dL. Technical criteria included CR after induction therapy for acute leukemia and less than 20% blasts for MDS. Patients older than 50, with a Hematopoietic Cell Transplantation (HCT)-specific comorbidity index above 3, or both, were ineligible for myeloablative conditioning (MAC) regimens [20]. If no MSD was available, the donor was a matched unrelated donor (MUD) or matched sibling donor (MSD). If no related or unrelated donor was available, haploidentical allo-SCT was used as an alternative.

The control group consisted of patients who did not receive PVP-ZG-TAU gel and who had the same eligibility characteristics as the experimental group. These patients underwent allogeneic transplantation consecutively in the year prior to the start of the study.

Quality control procedures were guaranteed throughout the study as the hospital transplant unit and laboratory received accreditation from the Joint Accreditation Committee of the International Society for Cellular Therapy (ISCT) and the European Group for Blood and Marrow Transplantation (EBMT), better known as JACIE.

## 3. Treatment

The two arms of the study differed only in terms of whether PVP-ZG-TAU gel was administered prophylactically for mucositis. Regarding chemotherapy regimens, mucositis assessment, GVHD prophylaxis, anti-infective therapy, and supportive care, the approaches were identical across both study arms.

Patients received either a MAC or an RIC regimen. The MAC regimen included thiotepa (5 mg/kg/day for 2 days, for a total dose of 10 mg/kg), busulfan (3.2 mg/kg/day for 3 days, for a total dose of 9.6 mg/kg), and fludarabine. 30 mg/m^2^/day for five days (total dose: 150 mg/m^2^). The RIC regimen combined thiotepa, busulfan, and fludarabine, with a one-day reduction in thiotepa (total dose: 5 mg/kg) and busulfan (total dose: 6.4 mg/kg), while fludarabine dosage remained unchanged. Fludarabine was administered as a 0.5 h infusion per day. Thiotepa and busulfan were administered according to the instructions in the European Summary of Product Characteristics. To prevent seizures, phenytoin or a benzodiazepine was administered daily to all patients in the busulfan arm between days −5 and −2. Peripheral blood was used as the graft source for all patients. Prophylaxis for graft-versus-host disease (GvHD) was standardized in both groups. It consisted of ciclosporin, starting on day −1 at a dose of 5 mg/kg daily, which was then adjusted based on blood levels, as well as a short course of methotrexate (15 mg/m^2^ on day +1 and 10 mg/m^2^ on days +3, +6, and +11). All recipients of matched related or unrelated donor transplants additionally received anti-T-lymphocyte immune globulin (ATG Fresenius): 5 mg/kg on days −4, −3, and −1 for related donors, and 10 mg/kg on days −4, −3, and −1 for unrelated donors. Haploidentical allo-SCT recipients received cyclosporine beginning on day −1, cyclophosphamide at 50 mg/kg/day on days +3 and +4, and mycophenolate mofetil from days +5 to +35. Antibiotic prophylaxis with sulfamethoxazole and trimethoprim was administered twice daily until the day of allo-SCT. All patients received oral acyclovir at a dose of 800 mg twice daily from day +3 until approximately day +90 post-allo-SCT. Patients received letermovir 240 mg once daily from day 0 to day +28 after allo-SCT, continuing until day +100. Red blood cell (RBC) and platelet transfusions were administered to maintain hemoglobin levels of at least 8 mg/dL and platelet counts of at least 10 × 10^9^/L, or to treat symptomatic anemia or minimal mucocutaneous hemorrhagic syndrome. Intravenous hydration and electrolyte support were also provided. If fever and neutropenia (FN) occurred, blood and catheter-drawn cultures were ordered, and intravenous piperacillin/tazobactam was promptly started. However, antibiotic therapy was administered based on the results of blood cultures or the colonization status of microbiological surveillance swabs.

In both arms, OM intensity was evaluated using a 5-point scale recommended by the World Health Organization (WHO) [21]. The WHO scale is used to assess the severity of oral mucosal lesions in patients, particularly those undergoing cancer treatments like chemotherapy or radiation therapy. It ranges from 0 to 5:grade 0—No change, no lesion; grade 1—soreness, erythema, or mild soreness; able to eat solids; grade 2—erythema, ulcers, patient cannot tolerate solid diet but can swallow liquids; grade 3—ulcers with extensive erythema; patient cannot swallow solid or liquids; grade 4—alimentation is not possible; alimentation requires parenteral or enteral support; grade 5—death.

Pain was assessed using a Numeric Rating Scale (NRS), which is a tool where patient rates their pain intensity on a scale of 0 to 10, where 0 is “no pain” and 10 is “the worst pain imaginable”. The patient indicates a number that best reflects their current level of pain, and this score is used to measure and track pain levels.

Preventing oral mucositis in both groups of our study involved maintaining good oral hygiene, including brushing with a soft toothbrush 4 times a day, and avoiding substances that cause local irritation, such as alcoholic mouth rinses and acidic, salty, or dry foods. A systemic antifungal drug (fluconazole) was included as standard of care. In the experimental group, PVP-ZG-TAU gel was sprayed directly onto lesions. The directional nozzle helped reach all affected areas of the mouth. Patients received three sprays, three times a day, from the start of conditioning chemotherapy until day +15 after allo-SCT. Patients who experienced moderate or severe OM received parenteral or mixed nutrition until the complication resolved. To treat OM, patients were advised to use multipurpose mouthwashes and antifungals. Those who experienced mucositis-related pain were treated with paracetamol and opioid drugs (morphine and tramadol) for severe pain. Informed consent for inclusion in the European Society for Blood and Marrow Transplantation (EBMT) registry was obtained from all patients. This study used fully anonymized data extracted from clinical records. Anonymization was performed prior to analysis by removing all direct identifiers and any information that could enable indirect re-identification. In accordance with Recital 26 and Article 4(1) of the GDPR, anonymized data do not constitute personal data; therefore, no Ethics Committee approval was required. The study complies with the principles of the Declaration of Helsinki and applicable data protection regulations.

## 4. Statistical Analysis

The data are reported as the mean and standard deviation (SD), the median and interquartile range (IQR), and percentages. Comparisons between groups were performed using the Mann–Whitney test or chi-squared test, as appropriate. The Mann–Whitney test was also used for normally distributed variables because it is more conservative (see Table 1 and Supplementary Table S1). The frequency of mucositis resolution over time is expressed as the number of cases of resolution per 100 person-weeks. The clinical impact magnitude of the PVP-ZG-TAU treatment on mucositis resolution was expressed as the number needed to treat (NNT). Univariable correlates of the mucositis resolution incidence rate over time were identified using crude (univariable) Cox models. In multiple Cox models, we adjusted for PVP-ZG-TAU therapy, age, and gender, irrespective of statistical significance. We also adjusted for baseline characteristics associated with time to mucositis resolution or that differed between PVP-ZG-TAU-treated and untreated patients. The data were expressed as hazard ratios (HRs), 95% confidence intervals (CIs), and *p*-values in the Cox models. Data analysis was performed using SPSS for Windows (version 29, IBM, Chicago, IL, USA) and R (version 4.4.3, Copyright © 2025 The R Foundation for Statistical Computing).

## 5. Results

Based on physical examinations, we analyzed the intensity of oral mucosal inflammatory changes after chemotherapy using the four-level WHO classification. Changes were assessed after the transplant procedure in the entire study population, accounting for the type of preparatory therapy used for allo-SCT. The study population included 82 patients. Thirty-nine of these patients (48%) were treated with PVP-ZG-TAU (Table 1). Patients treated with PVP-ZG-TAU differed significantly from untreated patients in terms of transplant type, nutrition type, and median parenteral nutrition duration. Patients treated with PVP-ZG-TAU developed higher fever grades more frequently and experienced less BMI loss than untreated patients (Table 2). Interestingly, patients receiving PVP-ZG-TAU were less likely to report grade 2–3 diarrhea according to WHO criteria (*p* = 0.084) than the remaining patients (Table 2). All patients had mucositis (see Table 1), and approximately one quarter of them (23%) developed grade 3–4 mucositis. The main characteristics of patients by mucositis severity (grade 3–4 versus grade 1–2) are provided in Supplementary Table S1.

### Cox Regression Analysis of Mucositis Resolution

Patients in both groups experienced resolution of mucositis over time. However, the incidence rate of mucositis resolution was higher in patients treated with PVP-ZG-TAU (84 resolutions per 100 person-weeks) than in untreated patients (62 resolutions per 100 person-weeks). This implies that for every five patients treated with PVP-ZG-TAU, one additional patient experiences mucositis resolution compared to the control group. Furthermore, patients treated with PVP-ZG-TAU had a 62% higher probability of earlier mucositis resolution than untreated patients (HR: 1.62, 95% confidence interval [CI]: 1.03–2.57), and this difference was statistically significant (*p* = 0.038). Similar analyses revealed that diagnosis type (MDS versus Leukemia) was also a significant predictor of earlier mucositis resolution (HR: 2.18; 95% confidence interval [CI]: 1.18–4.03; *p* = 0.013; see Table 3a). Notably, adjusting the data for potential confounders further amplified the effect of PVP-ZG-TAU on time to mucositis resolution (see Table 3b). After adjusting for confounders, the probability of earlier mucositis resolution was 68% higher in patients receiving PVP-ZG-TAU treatment than in untreated patients (HR: 1.68, 95% confidence interval [CI]: 1.03–2.74, *p* = 0.036). In the same multivariable model, diagnosis type (MDS versus Leukemia) was confirmed as an independent correlate of earlier mucositis resolution over time (HR: 2.39, 95% confidence interval [CI]: 1.20–4.73, *p* = 0.013).

## 6. Discussion

OM is the most common toxicity of allo-SCT [22,23,24]. Risk factors have historically been attributed to therapy and patient characteristics [1,22,23,24]. Various interventions have been attempted to prevent and manage mucositis, but there is limited robust evidence supporting their routine use [14,25,26]. Basic oral care (BaOrCa) is an interesting best practice that involves routine procedures to reduce bacterial loads in the oral cavity, prevent infections, and provide comfort during allo-SCT [26]. This strategy consists of multi-agent combination protocols involving bland mouth rinses, flossing, toothbrushes, and diluted sodium bicarbonate mouthwash. It is compared with other bland rinses, chlorhexidine (CHX) rinses, saline rinses, and other active-agent rinses. However, due to limited and inconsistent data, no guideline could be established regarding their use.

Data on anti-inflammatory agents such as benzydamine [27], celecoxib [28], irsogladine maleate [29], misoprostol [30], and rebamipide [31] have been published [32]. The evidence for each of these agents was insufficient to support their use in the transplant setting.

The rapidly growing field of laser and light therapy, which uses low-level energy to stimulate biological responses, is called photobiomodulation (PBM) [33]. The current guideline recommends preventing OM with intraoral PBM using low-level laser therapy in adult patients receiving a transplant conditioned with high-dose chemotherapy [34].

Cryotherapy causes vasoconstriction of the superficial blood vessels, limiting the delivery of cytotoxic drugs to oral tissue and reducing damage to the oral mucosa [35]. Since cooling is temporary, this treatment applies only to cytotoxic agents with a short half-life, such as melphalan [36,37].

Some trials tested keratinocyte growth factor 1 (KGF; palifermin) [38,39,40]. However, routine adoption in clinical practice is uncommon, which may reflect the lack of evidence justifying its use and the drug’s high cost [41].

The effects of various vitamins, minerals, and nutritional supplements were assessed [14,26,32]. These supplements included glutamine, an elemental diet, zinc, a supersaturated calcium phosphate rinse, vitamin E, selenium, folinic acid, and calcitriol. It was determined that parenteral glutamine should not be used to prevent OM in patients undergoing transplantation. New evidence was identified for the following: morphine (topical), sucralfate (topical/systemic), fluconazole (systemic), miconazole (topical and systemic), mucoadhesive hydrogel (topical), doxepin (topical), and fentanyl (transdermal).

PVP-ZG-TAU gel effectively addresses mineral deficiencies and inflammatory processes associated with oral pain and discomfort [15,16,17]. It has antimicrobial, anti-inflammatory, free radical scavenging, and analgesic properties. PVP-ZG-TAU delays the onset of mucositis by inhibiting the inflammatory cycle and providing pain relief by blocking COX-2-mediated prostaglandin production. Its biomechanical benefits include a bioadhesive barrier that provides durable protection. It blocks exposed nerve endings, coats lesions, and provides soothing relief. The gel also protects ulcerated tissue from further injury and bacterial colonization. PVP-ZG-TAU adheres to the mucosal surface and delivers a bioactive zinc-taurine complex that is released into epithelial cells, providing anti-inflammatory benefits. In vitro studies have demonstrated the synergistic anti-inflammatory effects of zinc and taurine in regulating COX-2-mediated prostaglandin production and inflammatory cytokine levels.

Colita et al. [17] evaluated the efficacy of PVP-ZG-TAU gel for prophylactic or curative treatment in children undergoing chemotherapy for hematologic malignancies. In this study, the oral spray was administered prophylactically or curatively. There was no difference in the median tolerability value of the PVP-ZG-TAU gel between the two groups. In the prophylaxis group, there was a significant reduction in OM severity, resulting in improved quality of life, pain control, and oral intake.

Niculita et al. [42] evaluated the efficacy of PVP-ZG-TAU in patients with hematologic malignancies. This single-center, observational, retrospective study analyzed 145 patients, including 99 adults and 46 pediatric patients. All patients received PVP-ZG-TAU prophylactically for OM. PVP-ZG-TAU actively prevented OM and significantly alleviated OM symptoms in patients who had already developed OM. 99.3% of the total cohort experienced OM prevention or remission after receiving PVP-ZG-TAU.

Dragomir et al. [43] conducted a single-center, open-label study to evaluate the safety and efficacy of PVP-ZG-TAU in children undergoing high-risk mucotoxic chemotherapy. Fifteen patients aged 5–16 were included in the study. PVP-ZG-TAU was administered alongside other therapeutic interventions from the early stages of mucositis. With PVP-ZG-TAU treatment, OM severity improved, mucositis duration was shorter, and swallowing and eating difficulties were reduced. Pain decreased, and the severity of OM did not delay treatment. No neutropenia-related complications occurred.

Zannier et al. [44] conducted a study evaluating the impact of three products on OM outcomes in patients receiving radio- and chemotherapy for solid cancers. Sixty adult patients were randomized into three groups, balanced for tumor site and treatment. They were assigned to receive either PVP-ZG-TAU (Group 1), Episil Oral Solution (Group 2), or Gelclair Oral Gel (Group 3). The PVP-ZG-TAU group demonstrated better efficacy and a higher safety profile than the other two groups. This study shows that correctly using anti-mucositis products alongside an appropriate hygiene and diet regimen is effective in preventing OM, one of the most frequent side effects in patients undergoing chemotherapy. No adverse events relating to PVP-ZG-TAU have been reported.

In our study, all patients developed oral mucositis, with no observed difference in grade between the treated and untreated groups. The apparent lack of preventive efficacy for PVP-ZG-TAU may be attributable to a significant imbalance in transplant type, as approximately half of the PVP-ZG-TAU group underwent haploidentical transplantation, which is strongly associated with higher mucositis grades. Despite this baseline difference, the absence of increased OM grades in the treated group is encouraging; however, our study does not allow us to draw conclusions in this regard, and further investigation would be needed. The primary objective was to assess the treatment’s effectiveness in resolving mucositis. While OM was self-limiting in all patients, resolution occurred notably faster in the PVP-ZG-TAU group. The resolution rate was 84 per 100 person-weeks in treated patients compared to 62 per 100 person-weeks in the control group, indicating one additional resolution event for every five patients treated. This clinically meaningful benefit was corroborated by time-to-event analysis. Unadjusted Cox regression analysis demonstrated that PVP-ZG-TAU treatment was associated with a 62% higher probability of earlier mucositis resolution. After adjustment for confounders, the effect remained significant and was further strengthened. Additionally, the underlying hematological diagnosis emerged as an independent predictor of recovery, with patients diagnosed with myelodysplastic syndrome achieving resolution more than twice as quickly as those with leukemia in both unadjusted models.

Conditioning regimens cause catastrophic extracellular matrix (ECM) damage in multiple organs, particularly oral/GI mucosa, endothelium, and stromal tissues [45]. ECM degradation is the invisible driver of mucositis, vascular complications, GVHD, and delayed engraftment. Adults have compromised ECM repair that pediatric patients do not. Pediatric studies cannot predict adult benefit due to biological differences [46]. PVP-ZG directly targets both protection and repair of the ECM [47]. No current interventions address matrix integrity as a primary target. Success would revolutionize prevention across multiple complication domains. As the matrix structure can be compromised by chemical reactions and mechanical stress, it is important to address these vulnerabilities through further investigation. Future research could focus on optimizing formulation stability, identifying protective excipients, or modifying processing conditions to minimize degradation.

To improve the applicability of future studies investigating PVP-ZG, researchers may consider implementing randomized controlled trials involving larger samples across multiple institutions.

## 7. Conclusions

In summary, although PVP-ZG-TAU may not prevent the onset of oral mucositis, it significantly accelerates mucositis resolution. These findings underscore the potential of PVP-ZG-TAU as a supportive intervention to lessen the burden and duration of mucositis, particularly in vulnerable patient populations. However, the non-randomized design with imbalanced groups prevents firm conclusions. Nevertheless, the trial is limited by its small sample size, heterogeneity of the two study groups due to the lack of randomization, as well as by the adoption of a non-concurrent (historical) control arm. There remains an urgent need for clinical studies that can definitively establish the effectiveness of available treatments for oral mucositis. As the number of transplant procedures continues to rise, further investigation into oral mucositis is warranted.

## Figures and Tables

**Table 1:** Patients’ characteristics by PVP-ZG-TAU treatment.

	All (n = 82)	No PVP-ZG-TAU (n = 43)	Yes PVP-ZG-TAU (n = 39)	*p* Value
**Patients’ characteristics at the time of ASCT**				
**Male gender, %**	44; 53.7	23; 53.5	21; 53.8	ns
**Age, years**	51.3 (13.5)	52.8 (13)	49.6 (13.9)	ns
**Diagnosis**				
Acute myeloid leukemia, %	50; 61.0	26; 60.5	24; 61.5%	ns
Acute lymphoblastic leukemia, %	19; 23.2%	10; 23.3%	9; 23.1%	ns
Myelodysplastic syndrome, %	13; 15.9	7; 16.3	6; 15.4	ns
**Type of transplant**				
SIB-MUD, %	56; 68.3	35; 81.4	21; 53.8	0.007
APLO, %	26; 31.7	8; 18.6	18; 46.2	
**CMV Status Donor(D)/Receiving(R), %**				
D+/R−	6; 7.3	3; 7	3; 7.6	ns
D+/R+	52; 63.4	29; 67.4	23; 59	ns
D−/R−	3; 3.7	2; 4.7	1; 2.6	ns
D−/R+	21; 25.6	9; 20.9	12; 30.8	ns
**HSV Status**				
IgG positive, %	77; 93.9	40; 93.1	37; 94.8	ns
IgG negative, %	5; 6.1	3; 6.9	2; 5.2	ns
**Conditioning regimen**				
RIC, %	11; 13.4	8; 18.6	3; 7.7	0.148
MAC, %	71; 86.6	35; 81.4	36; 92.3	
**Mucositis incidence, %**				
**Mucositis grade**				
Grade 1–2, %	63; 76.8	34; 79.1	29; 74.4	0.614
Grade 3–4, %	19; 23.2	9; 20.9	10; 25.6	
**Therapy**				
**Nutrition**				
Normal, %	21; 25.6	20; 46.5	1; 2.6	<0.001
Parenteral or mixed, %	61; 74.4	23; 53.5	38; 97.4	
Median duration of parenteral nutrition (days)	14 (11–19)	12 (5–18)	15 (13–19)	0.018
Median maximum pain perceived (units)	1 (1–3)	1 (1–2)	2 (1–3)	0.151
**Analgesic therapy**				
No or paracetamol, %	57; 69.5	32; 74.4	25; 64.1	0.311
Opioids or mixed, %	25; 30.5	11; 25.6	14; 35.9	
Median duration of analgesic therapy (days)	5 (4–11)	5 (2–10)	6 (5–12)	0.343

ns: not significant.

**Table 2:** Patients’ Outcome data by PVP-ZG-TAU treatment.

	All (n = 82)	No PVP-ZG-TAU (n = 43)	Yes PVP-ZG-TAU (n = 39)	Between-Group Difference and 95% CI	*p* Value
**Efficacy/safety measurements**					
**Fever WHO grade**					
**0–1 grade, %**	39; 47.6	25; 58.1	14; 35.9	−22.2 (−43.3; −1.1)	0.044
**2–4 grade, %**	43; 52.4	18; 41.9	25; 64.1	22.2 (1.1; 43.3)	
**Causes of fever**					
**FUO, %**	37; 50	19; 47.5	18; 52.9	5.4 (−16.2; 27.0)	0.641
**Sepsi, FUO-spesi, %**	37; 50	21; 52.5	16; 47.1	−5.4 (−27.0; 16.2)	
**Diarrhea WHO grade**					
**0–1 grade, %**	60; 73.2	28; 65.1	32; 82.1	17.0 (−1.6; 35.6)	0.084
**2–3 grade, %**	22; 26.8	15; 34.9	7; 17.9	−17.0 (−35.6; 1.6)	
**Median BMI difference (%)**	−7.7 (from −12.1 to −4.9)	−10.2 (from −13.8 to −6.2)	−6.1 (from −9.1 to −2.8)	4.2 (from 1.3 to 7.0)	0.005

**Table 3:** Cox’s analyses of mucositis resolution. (**a**) Univariable Cox analyses. (**b**) Multivariable Cox analysis.

(**a**)		
	**HR (95% CI)**	***p*** **-Value**
PVP-ZG-TAU (yes vs. no)	1.62 (1.03–2.57)	0.038
Gender (M vs. F)	1.23 (0.79–1.92)	0.350
Age at transplant (years)	0.99 (0.97–1.01)	0.259
Diagnosis (Myelodysplastic Syndrome vs. Leukemia)	2.18 (1.18–4.03)	0.013
Type of transplant (APLO vs. SIB-MUD)	0.86 (0.54–1.38)	0.527
Conditioning regimen (MAC vs. RIC)	0.93 (0.48–1.83)	0.840
(**b**)		
	**HR (95% CI)**	***p*-Value**
PVP-ZG-TAU (yes vs. no)	1.68 (1.03–2.74)	0.036
Diagnosis (Myelodysplastic Syndrome vs. Leukemia)	2.39 (1.20–4.73)	0.013
Gender (M vs. F)	1.13 (0.72–1.79)	0.593
Age at transplant (years)	0.99 (0.97–1.00)	0.108
Type of transplant (APLO vs. SIB-MUD)	0.80 (0.47–1.36)	0.416

## Data Availability

The original contributions presented in this study are included in the article/Supplementary Materials. Further inquiries can be directed to the corresponding author(s).

## Supplementary Materials

The following supporting information can be downloaded at https://www.mdpi.com/article/10.3390/diseases13120408/s1, Table S1: Patients’ characteristics by grade of mucositis.
