# Supplementary material for: Efficacy of Polyvinylpyrrolidone–Zinc Gluconate and Taurine Gel in the Prophylaxis of Oral Mucositis in Adults Undergoing High-Dose Chemotherapy and Allogeneic Stem Cell Transplantation
Source: Diseases. 2025 Dec 18;13(12):408. doi: 10.3390/diseases13120408 (PMC12732068; doi:10.3390/diseases13120408)
Supplement: Supplementary file 1 [file diseases-13-00408-s001.zip › diseases-3987338-supplementary.pdf]

Supplementary TableS1: Patients' characteristics by grade of mucositis.

	Mucositis grade 1-2 (n=63)	Mucositis grade 3-4 (n=19)	p value
Patients characteristics at the time of ASCT			
Male gender	55.6	47.4	0.53
Age, year	51.3 ±13.8	51 ±12.6	0.775
Diagnosis			
Leukemia	82.5	89.5	0.468
Myelodysplastic syndrome	17.5	10.5	0.468
Type of transplant			
SIB-MUD	76.2	42.1	0.005
APLO	23.8	57.9	0.005
Conditioning regimen			
RIC	17.5	0	0.050
MAC	82.5	100	0.050
Therapy			
Nutrition			
Normal	31.7	5.3	0.020
Parenteral or mixed	68.3	94.7	0.020
**Duration of parenteral nutrition (median days)***	13.5 (10.5-18)	16 (13-19)	0.188
Maximum pain perceived (median)	1 (1-2)	3 (2-5)	<0.001
Analgesic therapy			
No or paracetamol	77.8	42.1	0.003
Opioids or mixed	22.2	57.9	0.003
Duration of analgesic therapy (median days) **	5 (2-10)	10 (5-14)	0.143

*Among patients who underwent parenteral nutrition

**Among patients who underwent analgesic therapy
